# Comparative Evaluation of Sealer Voids Using Single-Cone Obturation With Different Root Canal Sealers: An In Vitro Scanning Electron Microscopic Study

**DOI:** 10.7759/cureus.112947

**Published:** 2026-07-19

**Authors:** Rakhi Singh, Arwa Hussain Chaiwala, Mohan Kumar Reddy Sirigiri, Devna Sharma, Aby Kuruvilla, Rupak Kumar Dasarraju

**Affiliations:** 1 Department of Conservative Dentistry and Endodontics, Vananchal Dental College and Hospital, Garhwa, IND; 2 Department of Dentistry, Southern Illinois University School of Family and Community Medicine, Springfield, USA; 3 Department of Conservative Dentistry and Endodontics, CKS Theja Dental College and Hospital, Tirupati, IND; 4 Department of Conservative Dentistry and Endodontics, Faculty of Dental Sciences, Shree Guru Gobind Singh Tricentenary University, Gurugram, IND; 5 Department of Conservative Dentistry and Endodontics, Al-Azhar Dental College, Thodupuzha, IND; 6 Department of Pedodontics and Preventive Dentistry, Priyadarshini Dental College and Hospital, Pandur, IND

**Keywords:** bioceramics, gutta-percha, root canal obturation, root canal sealers, scanning electron microscopy

## Abstract

Introduction: An effective three-dimensional seal is essential for the long-term success of root canal treatment. The presence of sealer voids may compromise the integrity of obturation by permitting bacterial leakage and reinfection. Contemporary root canal sealers differ in their physicochemical and biological properties, which may influence their adaptation to the dentinal walls and the occurrence of voids. Therefore, evaluating the sealing quality of different sealers is important for selecting the appropriate obturation material. Therefore, the aim of this study was to evaluate and compare the sealer void area produced by BioRoot RCS (Septodont, Saint-Maur-des-Fossés, France), Sealapex (Kerr Corporation, Orange, CA, USA), and AH Plus (Dentsply DeTrey GmbH, Konstanz, Germany) following single-cone obturation using scanning electron microscopy (SEM).

Materials and methods: Forty-five extracted human single-rooted mandibular premolars were instrumented using the ProTaper Universal rotary system (Dentsply Sirona, Ballaigues, Switzerland) to size F3 and were randomly allocated into three groups (n = 15): BioRoot RCS, Sealapex, and AH Plus. Following standardized irrigation and single-cone obturation, all specimens were stored under humid conditions to allow for complete setting of the sealers. The roots were sectioned into apical, middle, and coronal thirds and examined using SEM. The sealer void area was measured for each section. Data normality was assessed using the Shapiro-Wilk test. Repeated-measures analysis of variance (ANOVA) was used for intragroup comparisons, whereas one-way and two-way ANOVA were performed for intergroup comparisons. Statistical significance was set at p < 0.05.

Results: Sealer voids were identified in all three experimental groups. The cumulative mean void area was the lowest for BioRoot RCS (0.0275 ± 0.0164 mm²), followed by AH Plus (0.0430 ± 0.0396 mm²), whereas Sealapex demonstrated the highest mean void area (0.0465 ± 0.0385 mm²). However, the intergroup comparison revealed no statistically significant difference among the three sealers (one-way ANOVA, p = 0.261, η² = 0.062). Intragroup analysis showed no significant variation in void distribution among the apical, middle, and coronal thirds for BioRoot RCS (p = 0.678), Sealapex (p = 0.984), and AH Plus (p = 0.229). Furthermore, two-way ANOVA demonstrated that neither the sealer type (p = 0.254, partial η² = 0.0215) nor the root canal level (p = 0.472, partial η² = 0.0119) significantly affected void formation. The interaction between sealer type and root canal level was also not significant (p = 0.553, partial η² = 0.0236), indicating comparable sealing performance among all the tested sealers.

Conclusion: Under the conditions of this in vitro study, BioRoot RCS, Sealapex, and AH Plus demonstrated comparable sealing performances when used with the single-cone obturation technique. Although minor variations in the mean void area were observed, these differences were not statistically significant. These findings suggest that all three sealers provide satisfactory adaptation to canal walls, and factors other than void formation may guide material selection in clinical practice.

## Introduction

Successful root canal treatment depends on the effective cleaning, shaping, and three-dimensional obturation of the root canal system. Obturation plays a critical role in preventing bacterial ingress and entombing residual microorganisms within the canal space [1]. An ideal obturation should establish a fluid-tight seal throughout the entire root canal system; however, anatomical complexities and material-related limitations often result in the formation of voids within the filling material or at the sealer-dentin interface [2]. These voids may serve as potential pathways for bacterial leakage, compromise the apical seal, and ultimately contribute to persistent periapical disease and endodontic failure [3].

The introduction of nickel-titanium rotary instrumentation has popularized the single-cone obturation technique because it is simple, reproducible, and well-matched to the tapered canal preparation produced by contemporary rotary systems [4]. The success of this technique largely depends on the sealing ability and physicochemical properties of the root canal sealer, as the sealer occupies the space between the gutta-percha and dentinal walls [5]. Consequently, the selection of an appropriate sealer has become increasingly important for achieving long-term treatment success.

Root canal sealers differ considerably in their composition, biological behavior, and anti-microbial efficacy [6]. AH Plus (Dentsply DeTrey GmbH, Konstanz, Germany), an epoxy resin-based sealer, has long been regarded as a reference material owing to its dimensional stability and favorable sealing characteristics. Sealapex (Kerr Corporation, Orange, CA, USA), a calcium hydroxide-based sealer, provides biological advantages through hydroxyl ion release but exhibits relatively higher solubility [7]. More recently, BioRoot RCS (Septodont, Saint-Maur-des-Fossés, France), a calcium silicate-based bioceramic sealer, has attracted considerable attention owing to its bioactivity, hydrophilicity, biocompatibility, and potential to form chemical bonds with dentin [8]. Despite these advances, none of the currently available sealers completely eliminates void formation, and the comparative evidence regarding their sealing performance remains inconsistent.

Scanning electron microscopy (SEM) permits high-resolution visualization of the sealer-dentin interface and enables precise assessment of sealer voids at different root levels [5]. Despite significant advances in root canal sealers, no material has consistently demonstrated the ability to produce void-free obturations. Variations in physicochemical properties, flow characteristics, adhesion to dentin, and setting behavior of different sealers may influence the quality of the apical seal and the occurrence of voids within the obturated canal. Furthermore, evidence comparing contemporary bioceramic, calcium hydroxide-, and epoxy resin-based sealers using the single-cone obturation technique remains limited and inconclusive. Therefore, the present in vitro study was undertaken using SEM to evaluate and compare the area and distribution of sealer voids produced by BioRoot RCS, Sealapex, and AH Plus following single-cone obturation. This study aimed to determine whether significant differences exist in sealer void formation among these materials and across the coronal, middle, and apical thirds of the root canal, thereby providing evidence of their sealing performance and potential clinical applicability.

## Materials and methods

This in vitro study was conducted in the Department of Conservative Dentistry and Endodontics, Vananchal Dental College and Hospital, Garhwa, India, between March 2023 and September 2023, after obtaining a waiver from the institutional ethics committee (approval number: IEC/VDC/2023/07-02). 45 freshly extracted human single-rooted mandibular premolars, for orthodontic or periodontal reasons, were included in the study. Teeth exhibiting fully formed apices, a single straight canal, and an intact root morphology were selected. Teeth with caries, restorations, root fractures, developmental anomalies, internal or external resorption, previous endodontic treatment, calcified canals, or open apices were excluded from the study. Following extraction, adherent soft tissue and calculi were removed using hand scalers, and the teeth were disinfected in 0.1% thymol solution for 24 hours before storage in distilled water at room temperature until use. The storage solution was periodically replaced to prevent dehydration of the specimens.

The sample size was estimated a priori using G*Power software version 3.1.9.7 (Heinrich Heine University Düsseldorf, Düsseldorf, Germany) for an F-test comparing three independent experimental groups. Based on a previously published study evaluating void formation among root canal sealers, an effect size (Cohen's f) of 0.60, alpha error probability of 0.05, and statistical power of 80% were selected [9]. The calculated minimum sample size was 45 specimens, corresponding to 15 teeth in each experimental group, which was considered adequate to detect statistically significant differences between the groups.

Before the commencement of the experiment, the principal investigator calibrated the specimen preparation, obturation, specimen sectioning, and scanning electron microscopic evaluation under the supervision of an endodontist with more than 10 years of clinical and research experience. Ten extracted teeth that were not included in the final sample were used for operator training and standardization of all experimental procedures. To evaluate measurement reproducibility, sealer void measurements from the pilot specimens were repeated after a two-week interval under identical operating conditions. Intra-examiner reliability was assessed using the intraclass correlation coefficient (ICC), which demonstrated excellent agreement (ICC = 0.92), confirming the satisfactory reproducibility of the measurements before the commencement of the study.

Following specimen selection, crowns were accessed using a round diamond bur (Dentsply Sirona, Ballaigues, Switzerland) in a high-speed air turbine handpiece (NSK Nakanishi Inc., Kanuma, Tochigi, Japan). Canal patency was confirmed using a size #15 K-file (MANI Inc., Utsunomiya, Tochigi, Japan). The working length was established electronically using an apex locator (Woodpecker Medical Instrument Co., Guilin, Guangxi, China) and confirmed radiographically by positioning the file 1 mm short of the apical foramen. A single calibrated operator performed all procedures to eliminate inter-operator variability.

Root canal preparation was performed using the ProTaper Universal nickel-titanium rotary system (Dentsply Sirona, Ballaigues, Switzerland) operated with an X-Smart endodontic motor (Dentsply Sirona, Ballaigues, Switzerland) following the crown-down technique until the F3 file was finished. Throughout instrumentation, the canals were irrigated with 5.25% sodium hypochlorite solution (Maarc Dental, Lucknow, Uttar Pradesh, India) delivered through a 27-gauge side-vented irrigation needle (Nipro Corporation, Osaka, Japan). After instrumentation was completed, smear layer removal was achieved using 17% ethylenediaminetetraacetic acid (EDTA) (Avue Prep+, Avue Technologies, Gurugram, India) for one minute, followed by a final rinse with sterile saline (Nirlife Healthcare Ltd., Ahmedabad, Gujarat, India). The canals were subsequently dried with sterile absorbent paper points corresponding to F3 preparation.

The specimens were randomly allocated to three equal groups (n = 15 each) using a computer-generated randomization sequence. Group I was obtained using a BioRoot RCS bioceramic sealer with a matched F3 gutta-percha cone (DiaDent Group International, Burnaby, British Columbia, Canada). Group II received a sealer based on a sealer (Kerr Corporation, Orange, California, USA) with an F3 gutta-percha cone, whereas Group III was obtained using AH Plus epoxy resin-based sealer with an identical gutta-percha cone. The assigned sealer was introduced into the canal using a size #25 Lentulo spiral (MANI Inc., Utsunomiya, Tochigi, Japan) operated at a low speed to ensure uniform coating of the canal walls.

A matched F3 gutta-percha master cone exhibiting definite tug-back at the working length was coated with the corresponding sealer and slowly inserted into the full working length using the single-cone obturation technique. Excess gutta-percha was removed using a heated instrument, and the coronal portion was vertically compacted using a hand plugger. The access cavities were temporarily sealed, and all specimens were stored at 37°C in 100% humidity for seven days to allow complete setting of the sealers.

Following complete setting, each specimen was decoronated at the cementoenamel junction using a water-cooled diamond disc (DFS Diamon GmbH, Riedenburg, Germany). Standardized markings were made at 1, 5, 9, and 13 mm from the anatomical apex using a digital Vernier caliper (Breeze Tools, India). Each root was sectioned perpendicular to its long axis to obtain representative slices corresponding to the apical, middle, and coronal thirds. The sectioned specimens were dehydrated, mounted on aluminum stubs, sputter-coated with platinum, and examined under a scanning electron microscope (JSM-6390LV; JEOL Ltd., Akishima, Tokyo, Japan). Representative images were obtained at standardized magnification for all specimens under identical operating conditions. Cross-sectional images were analyzed to identify and quantify sealer voids present between the obturation material and dentinal walls as well as within the sealer mass. The total void area for each section was measured in square millimeters using calibrated image analysis procedures, and the mean values for the apical, middle, coronal, and cumulative root thirds were recorded for statistical analysis.

All measurements were performed independently by a calibrated examiner who was blinded to the group allocation throughout the image analysis. To determine the measurement reliability, 20% of the scanning electron microscopic images were randomly selected and re-evaluated after two weeks under identical conditions. Intra-examiner agreement was assessed using the ICC, and an ICC value greater than 0.90 was considered indicative of excellent reproducibility.

Statistical analyses were performed (IBM SPSS Statistics version 25.0, IBM Corp., Armonk, New York, USA). Data normality was assessed using the Shapiro-Wilk test, which confirmed the normal distribution of all continuous variables (p > 0.05). Therefore, parametric statistical methods were employed in the analysis. Intragroup comparisons of void areas among the apical, middle, and coronal thirds were performed using repeated-measures analysis of variance (ANOVA). Intergroup comparisons of the cumulative void area among BioRoot RCS, Sealapex, and AH Plus were performed using one-way ANOVA followed by Tukey's honestly significant difference (HSD) post hoc test when appropriate. Additionally, two-way ANOVA was used to evaluate the independent effects of sealer type, root canal level, and their interaction on void area. Continuous variables were expressed as mean ± standard deviation, effect sizes were reported as eta-squared (η²) or partial eta-squared, where appropriate, and statistical significance was established at p < 0.05.

## Results

**Figure 1:**
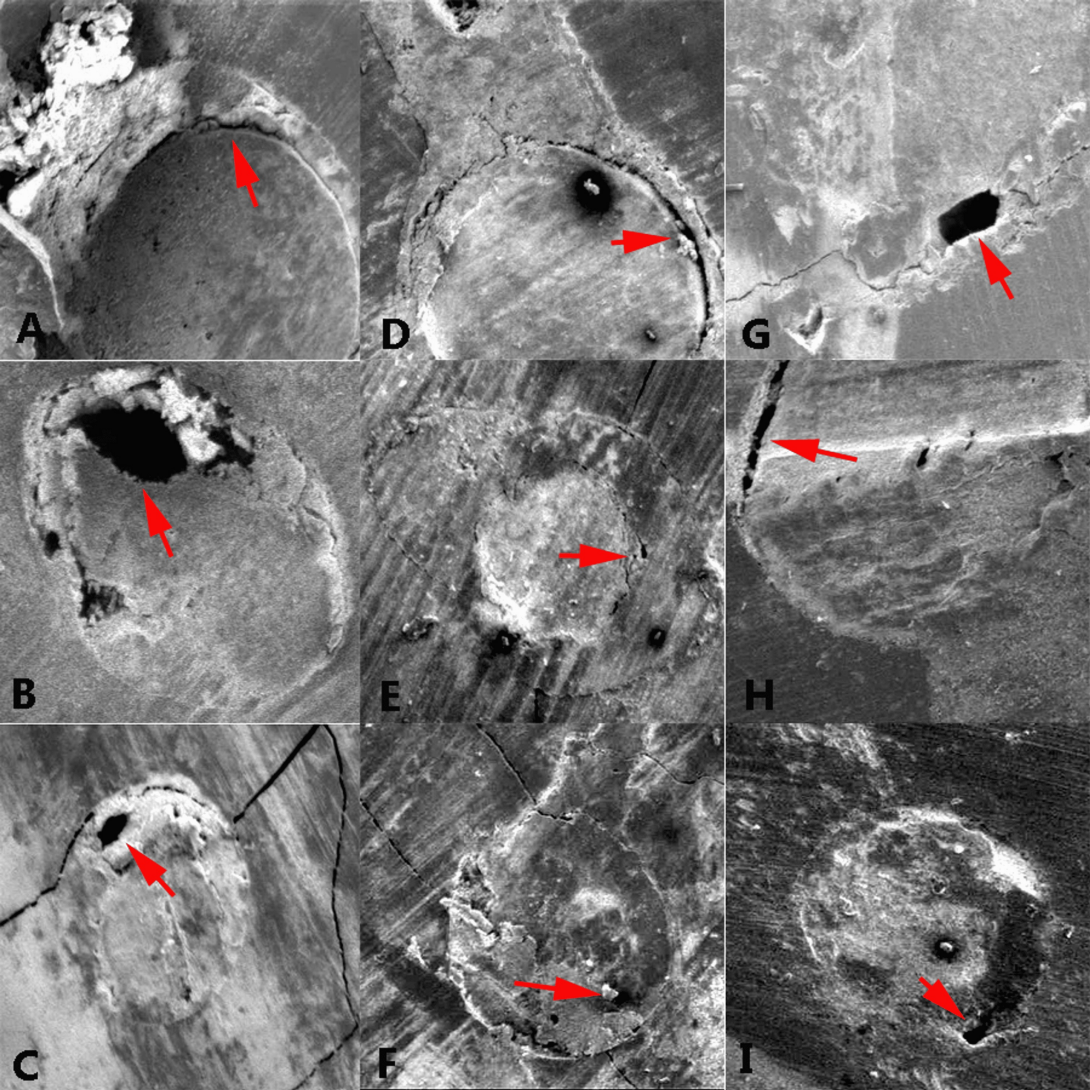
Representative scanning electron microscopic (SEM) images showing sealer voids (red arrows) at the sealer-dentin interface and within the obturated root canal following single-cone obturation with the three experimental sealers: (A-C) BioRoot RCS group (Group I), (D-F) Sealapex group (Group II), and (G-I) AH Plus group (Group III). The red arrows indicate representative sealer voids observed at different root canal levels, demonstrating variations in the location and morphology of voids among the tested sealers. All images were obtained under standardized SEM operating conditions. Original magnification: ×85; scale bar = 200 μm

A total of 45 extracted human mandibular premolars were included in the study and were equally distributed among the three experimental groups (15 specimens per group). SEM demonstrated the presence of sealer voids in all three groups, although the extent and distribution varied among the different sealers (Figure 1). Voids were observed both at the sealer-dentin interface and within the sealer mass. Although minor variations in the size and distribution of voids were evident among BioRoot RCS, Sealapex, and AH Plus, no consistent qualitative differences were observed, which corroborated the quantitative analysis showing no statistically significant differences among the tested sealers.

Intragroup comparison of void area across the apical, middle, and coronal thirds revealed no statistically significant differences among any of the three sealers (Table 1). BioRoot RCS demonstrated a relatively consistent void distribution throughout the root canal (p = 0.678), while Sealapex (p = 0.984) and AH Plus (p = 0.229) also exhibited comparable void areas across the three root levels. The corresponding effect sizes were small, indicating that the root canal level had a minimal influence on void formation within each sealer group.

**Table 1:** Intragroup comparison of sealer void area at different root canal levels within each experimental group. Data are presented as mean ± standard deviation (SD), repeated-measures analysis of variance (ANOVA) was used for intragroup comparison, η² indicates effect size, p < 0.05 was considered statistically significant.

Group	N	Apical third (mm²)	Middle third (mm²)	Coronal third (mm²)	Repeated-measures ANOVA F value	p-value	Effect size (η²)
		Mean ± SD	Mean ± SD	Mean ± SD			
Bioroot RCS	15	0.0098 ± 0.0087	0.0074 ± 0.0078	0.0103 ± 0.0115	0.39	0.678	0.019
Sealapex	15	0.0161 ± 0.0206	0.0147 ± 0.0219	0.0157 ± 0.0241	0.01	0.984	0.001
AH Plus	15	0.0067 ± 0.0134	0.0151 ± 0.0223	0.0212 ± 0.0298	1.52	0.229	0.068

The two-way ANOVA demonstrated that neither the type of root canal sealer nor the root canal level significantly influenced the measured void area (Table 2). Furthermore, no significant interaction was observed between sealer type and canal level (all p > 0.05), suggesting that the pattern of void formation remained similar among the three sealers, irrespective of the evaluated root third.

**Table 2:** Two-way analysis of variance (ANOVA) showing the effects of sealer type, root canal level, and their interaction on sealer void area. Two-way ANOVA was used to evaluate the main effects of sealer type and root canal level and their interaction; partial η² represents effect size, p < 0.05 was considered statistically significant; df: degrees of freedom.

Source	Sum of squares	df	Mean square	F value	p‑value	Partial η²
Sealer (BioRoot/Sealapex/AH Plus)	0.0010224	2	0.0005112	1.386	0.254	0.0215
Position (Apical/Middle/Coronal)	0.0005571	2	0.0002786	0.755	0.472	0.0119
Sealer × Position (Interaction)	0.0011208	4	0.0002802	0.760	0.553	0.0236

Intergroup comparison of the cumulative void area also showed no statistically significant differences between BioRoot RCS, Sealapex, and AH Plus (Table 3). Although BioRoot RCS demonstrated the lowest mean cumulative void area, and Sealapex showed the highest, these differences were not statistically significant (one-way ANOVA, p = 0.261). The observed effect size was moderate but indicated a comparable overall sealing performance among the three evaluated sealers. 

**Table 3:** Intergroup comparison of cumulative sealer void area among BioRoot RCS, Sealapex, and AH Plus. Data are presented as mean ± standard deviation (SD), one-way analysis of variance (ANOVA) was used for intergroup comparison, η² indicates effect size, p < 0.05 was considered statistically significant.

Group	BioRoot RCS	Sealapex	AH Plus	One-way ANOVA (F)	p-value	Effect size
	Mean ± SD	Mean ± SD	Mean ± SD			
Cumulative Void Area (mm²)	0.0275 ± 0.0164	0.0465 ± 0.0385	0.0430 ± 0.0396	1.387	0.261	0.062

## Discussion

The quality of root canal obturation is a major determinant of endodontic treatment success because an effective three-dimensional seal prevents the ingress of microorganisms and prevents any residual bacteria within the root canal system [1]. The presence of voids within the sealer or at the sealer-dentin interface may compromise the sealing ability of the obturation and provide potential pathways for bacterial leakage, ultimately contributing to post-treatment disease [2,3]. Consequently, the present in vitro study compared the sealer void areas produced by BioRoot RCS, Sealapex, and AH Plus following single-cone obturation using SEM.

The principal finding of the present study was that all three root canal sealers exhibited sealer voids, and no statistically significant differences were observed among the three materials or across the apical, middle, and coronal thirds of the root canal. Although BioRoot RCS demonstrated the lowest cumulative mean void area and Sealapex exhibited comparatively greater void formation, these differences were not statistically significant. These findings indicate that under standardized instrumentation and obturation conditions, the three sealers provided comparable adaptation to canal walls when used with the single-cone obturation technique [10].

The absence of significant differences among the tested sealers may be attributed to the standardized methodology employed in the present study. All specimens underwent identical canal preparation using the ProTaper Universal rotary system to the F3 preparation, were irrigated using a uniform protocol, and were obturated using matched-taper gutta-percha cones by a single calibrated operator. Such standardization minimizes procedural variability and suggests that the physicochemical differences among the sealers are insufficient to produce clinically meaningful variations in void formation under controlled laboratory conditions. Furthermore, the matched taper obtained with contemporary rotary instrumentation may reduce the thickness of the sealer layer, thereby decreasing the likelihood of void formation, irrespective of the sealer composition.

BioRoot RCS had the lowest mean cumulative void area, although the difference was not statistically significant. This observation may be explained by the calcium silicate composition, hydrophilic nature, and slight setting expansion of the material, which have been reported to improve adaptation to dentinal walls [11]. In addition, the release of calcium ions and the formation of hydroxyapatite at the dentin-sealer interface may enhance marginal adaptation over time [12]. However, despite these favorable biological characteristics, the present findings suggest that these advantages did not translate into a significantly lower incidence of voids immediately after obturation.

AH Plus also demonstrated favorable sealing performance with relatively low void formation. Its established dimensional stability, excellent flow characteristics, low solubility, and strong adhesion to dentin have consistently contributed to its reputation as a reference standard among resin-based sealers [7]. The present findings further support previous investigations demonstrating that AH Plus continues to provide a reliable seal when used with the single-cone obturation technique [13,14].

Sealapex exhibited comparatively higher mean void values than those of the other two materials; however, these differences were not statistically significant. Calcium hydroxide-based sealers possess biological advantages through hydroxyl ion release and stimulation of mineralized tissue formation but are also characterized by greater solubility and dimensional changes during setting [7]. These characteristics may contribute to the slightly increased void formation. Nevertheless, the magnitude of the differences observed in the present study was small and unlikely to be clinically relevant.

The present findings are consistent with those reported by Viapiana et al. [11], who compared BioRoot RCS and AH Plus using micro-computed tomography and fluid transport analysis and observed no significant difference in the overall sealing ability despite minor differences in void volume. Similarly, Huang et al. [13] demonstrated that neither the EndoSequence BC Sealer nor AH Plus produced completely void-free obturations when evaluated using micro-computed tomography and scanning electron microscopy, concluding that both sealers exhibited comparable sealing performance. Likewise, Atmeh et al. [15] reported that calcium silicate-based sealers continued to exhibit voids after aging, indicating that the complete elimination of voids remains difficult regardless of the sealer used. Collectively, these observations support the findings of the present study that void formation is an inherent characteristic of contemporary obturation techniques.

Conversely, several previous investigations have reported the superior sealing performance of bioceramic sealers. Kaul et al. [16] observed better adaptation of EndoSequence BC sealer than AH Plus using SEM, whereas Asawaworarit et al. [17] reported improved sealing ability of tricalcium silicate-based sealers compared with resin-based sealers. Similarly, Vijith et al. [18] demonstrated superior apical sealing of BioRoot RCS compared with AH Plus. The discrepancy between these findings and those of the present study may be explained by differences in study design, evaluation techniques, specimen selection, canal morphology, obturation protocols, and methods used for quantifying voids. Studies employing micro-computed tomography evaluate the entire obturation volume three-dimensionally, whereas the present investigation used SEM on standardized root sections, which provides excellent surface resolution but evaluates selected cross-sectional areas rather than complete root filling.

Another important finding of the present study was the absence of statistically significant differences between the apical, middle, and coronal thirds within each sealer group. This suggests that the standardized single-cone obturation technique produced a relatively uniform sealer adaptation throughout the prepared canal [19]. Although anatomical complexity generally increases toward the apical region, the matched taper produced by rotary instrumentation and careful sealer placement using a lentulo spiral may have contributed to the homogeneous distribution of the obturation material.

From a clinical perspective, the present findings indicate that BioRoot RCS, Sealapex, and AH Plus can achieve satisfactory adaptation when used with an appropriately standardized single-cone obturation protocol. Since no significant differences in void formation were observed, clinicians may select a sealer based on other clinically relevant properties such as biocompatibility, bioactivity, retreatability, handling characteristics, setting behavior, and cost, rather than void formation alone.

Certain limitations of this study should be acknowledged when interpreting the present findings. This study was performed under controlled in vitro conditions that cannot completely reproduce the complex biological environment of the root canal system. Only straight single-rooted mandibular premolars were evaluated, and the findings may not be directly applicable to teeth with a complex canal anatomy. In addition, SEM evaluates selected cross-sectional sections rather than the complete three-dimensional obturation volume. Future investigations employing micro-CT, larger sample sizes, artificial aging protocols, thermocycling, and bacterial leakage models would provide a more comprehensive understanding of the long-term sealing performance of contemporary root canal sealers.

## Conclusions

Within the limitations of this in vitro study, BioRoot RCS, Sealapex, and AH Plus demonstrated comparable sealing performances when used with the single-cone obturation technique. Although BioRoot RCS exhibited the lowest mean cumulative void area, and Sealapex showed comparatively greater void formation, these differences were not statistically significant. Furthermore, void distribution remained similar across the apical, middle, and coronal thirds. These findings suggest that all three sealers provide clinically acceptable adaptation under standardized obturation conditions, and sealer selection should also consider the biological properties, handling characteristics, and long-term clinical performance.
